# Biallelic mutations in the gene encoding eEF1A2 cause seizures and sudden death in F0 mice

**DOI:** 10.1038/srep46019

**Published:** 2017-04-05

**Authors:** Faith C. J. Davies, Jilly E. Hope, Fiona McLachlan, Francis Nunez, Jennifer Doig, Hemant Bengani, Colin Smith, Catherine M. Abbott

**Affiliations:** 1 Centre for Genomic & Experimental Medicine, MRC Institute of Genetics and Molecular Medicine, University of Edinburgh, Western General Hospital, Crewe Road, Edinburgh, EH4 2XU, United Kingdom; 2 Muir Maxwell Epilepsy Centre, University of Edinburgh, 20 Sylvan Place, Edinburgh, EH9 1UW, United Kingdom; 3 MRC Human Genetics Unit, MRC Institute of Genetics and Molecular Medicine, University of Edinburgh, Western General Hospital, Crewe Road, Edinburgh, EH4 2XU, United Kingdom; 4 Academic Department of Neuropathology, Centre for Clinical Brain Sciences, Chancellor’s Building, Little France, Edinburgh, EH16 4SB, United Kingdom

## Abstract

*De novo* heterozygous missense mutations in the gene encoding translation elongation factor eEF1A2 have recently been found to give rise to neurodevelopmental disorders. Children with mutations in this gene have developmental delay, epilepsy, intellectual disability and often autism; the most frequently occurring mutation is G70S. It has been known for many years that complete loss of eEF1A2 in mice causes motor neuron degeneration and early death; on the other hand heterozygous null mice are apparently normal. We have used CRISPR/Cas9 gene editing in the mouse to mutate the gene encoding eEF1A2, obtaining a high frequency of biallelic mutations. Whilst many of the resulting founder (F0) mice developed motor neuron degeneration, others displayed phenotypes consistent with a severe neurodevelopmental disorder, including sudden unexplained deaths and audiogenic seizures. The presence of G70S protein was not sufficient to protect mice from neurodegeneration in G70S/− mice, showing that the mutant protein is essentially non-functional.

Exome sequencing of parent-child trios has uncovered single gene *de novo* mutations underlying many previously unexplained cases of severe neurodevelopmental disorders. In many cases the genes involved have previously been implicated in familial forms of the disorders, but in others, mutations in novel causative genes have been uncovered. One such gene is *EEF1A2*, which encodes translation elongation factor 1A2 or eEF1A2.

Translation elongation is the step in protein synthesis in which aminoacylated tRNAs are delivered to the ribosome. This process is facilitated by the elongation factor eEF1A, which delivers the aa-tRNA to the A site of the ribosome in a GTP-dependent process. Complete loss of eEF1A leads to the failure of *de novo* protein synthesis. In vertebrates, there are two independently encoded isoforms of eEF1A, called eEF1A1 and eEF1A2^1^,^2^. Translation elongation factor eEF1A2 is expressed in a very restricted pattern, with expression only being found in neurons and muscle (skeletal and cardiac). Other tissues express eEF1A1, which is also present in neurons and muscle during development. In mice, eEF1A1 is downregulated after birth until by 21 days, just after weaning, it is no longer detectable in muscle, heart and neurons^3^,^4^. In adult organisms the two isoforms are mutually exclusively expressed, suggesting functionally equivalent roles in protein synthesis^5^; this is borne out by the finding that either can support translation in *in vitro* assays^6^.

A spontaneously occurring deletion of eEF1A2 in mice causes early onset neurodegeneration in mice when the mutation is homozygous. This mutation, called wasted (gene symbol *wst*) results in complete ablation of expression of eEF1A2, and homozygous mice die by 28 days at the latest with motor neuron degeneration and concomitant muscle wasting. This phenotype is not ameliorated by the forced expression of eEF1A2 in muscle, suggesting that the primary lesion occurs in neurons^3^,^7^. Mice that are heterozygous for the wasted deletion mutation, on the other hand, not only grow and breed normally, but also show no sign of neurodegeneration or impairment of motor function even when aged to 21 months^8^.

Heterozygous missense mutations in *EEF1A2* have now been described in at least 17 individuals^9^,^10^,^11^,^12^,^13^. In almost all cases these individuals have severe epilepsy, many have autism, and all have developmental delay and intellectual disability. In the most extreme cases the children can be wheelchair bound and unable to make purposeful movements. Thus far 8 distinct amino acid substitutions have been identified. In each case, the mutation is seen in the affected child but not in the parents. In such cases, where mutations have arisen *de novo* and there is no inheritance pattern, there is a significant burden of proof that the mutations are actually causative. Clearly, the more mutations that are uncovered in a given gene and associated with a specific disorder, the more likely they are to be causative. Even so, it is a crucial step towards therapy to determine the mechanism by which the mutations cause dysfunction, particularly in the case of heterozygous, dominant mutations.

We carried out experiments to address the effects of disease-causing missense mutations on the function of eEF1A2. We used CRISPR/Cas9 genome editing to create mice carrying the G70S mutation, the mutation that has been identified in the largest number of patients. Our intention had been to set up a breeding line of mice carrying the G70S mutation, but the high frequency of biallelic mutations seen in the gene encoding eEF1A2 meant that none of the mice carrying the missense mutation survived past 4 weeks. We therefore analysed the F0 founder mice; the limitations of this approach, including mosaicism, are addressed further in the discussion.

Many of the mice had biallelic deletions leading to complete loss of function, but even though they had a characteristic wasted neurodegenerative phenotype, we also observed seizures and sudden deaths in these mice. One mouse was homozygous for the G70S mutation and was more severely affected than the mice with complete null mutations. On the other hand, we show that the presence of eEF1A2 carrying the G70S mutation (in the absence of wild-type eEF1A2) is insufficient to prevent neurodegeneration, suggesting that the G70S protein is unable to function normally in protein synthesis. These observations, in combination with the lack of seizures seen in heterozygous null mice, suggest that there is both a loss of function and dominant negative/gain of function at play in the children with missense mutations in eEF1A2.

## Results

### CRISPR/Cas9 gene editing design

**Figure 1:**
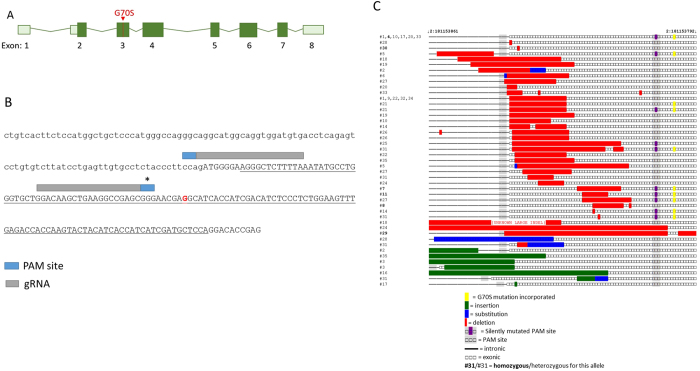
CRISPR/Cas9 experimental design and results of genotyping of F0 animals. (**A**) Diagrammatic representation of mouse *Eef1a2* gene showing the position of the G70S mutation in exon 3 of *Eef1a2*. (**B**) Sequence of *Eef1a2* in the region targeted in the CRISPR/Cas9 experiment. Underlined sequence indicates the ssODN repair template, lower case indicates intron sequence, upper case is coding sequence. Letter in bold red indicates the site of the G70S mutation (changed to A in repair template). *Indicates PAM site (protospacer adjacent motif), targeted by the gRNA, that was mutated in the template to GTG to prevent further targeting. (**C**) Sequences of alleles recovered in all mice analysed, organised according to the location of the mutation. The number on the left corresponds to the mouse code number shown in Supplementary Table 1. Colour coding indicates the nature of the mutation.

We sought to recreate the mouse equivalent of the clinically important G70S mutation in mice. We used CRISPR/Cas9 genome editing; paired gRNAs that target the *Eef1a2* gene in the region of the mutation, a single stranded oligonucleotide (ssODN) repair template containing the mutation, and Cas9 nickase RNA were injected into single cell mouse embryos. The gRNA pair, target region and repair template design used are shown in Fig. 1A and B.

The G70S mutation was selected since, at the time, it was the only mutation to have arisen independently three times. However, the position of this mutation placed constraints on the design of the ssODN repair template. Ideally, the NGG sites targeted by the gRNAs, the so-called “PAM” (protospacer adjacent motif) sites, would be mutated to non-NGG sequences in the repair template, preventing subsequent rounds of mutation in the developing mouse embryo that could lead to unwanted deletions. We were able to engineer a change in the site targeted by the 3′ gRNA, introducing a silent substitution in order to prevent further mutations after the repair template had been incorporated (Fig. 1B). However, the upstream PAM site, targeted by the 5′ gRNA, was too close to a splice site to be able to introduce a further mutation without risking unpredicted effects on splicing and gene expression. This site was therefore left unmutated in the repair template.

### Genotyping shows specific mutations in *Eef1a2* in almost all mice

Thirty five mice were born after two rounds of injection of the gRNAs, repair template and Cas9n mRNA into fertilised oocytes. DNA was purified from ear notches of the mice and the region around the predicted mutation site, spanning both PAM sites, was amplified and sequenced. In cases where more than two alleles appeared to be present, or where there was any ambiguity in the sequence analysis, the PCR products were cloned and individual clones resequenced. Further clarification was obtained where possible by designing allele-specific PCR assays (for example where a primer binding site was deleted in one allele, forcing amplification of only the other allele) and sequencing the product (Supplementary Figure 1).

Analysis of DNA from the mice showed a variety of different mutations occurring in the region targeted by the gRNAs (Fig. 1C, Table 1 and Supplementary Table 1). Only two mice of the 35 born had two wild-type alleles (#s 15 and 23). Four mice could confidently be characterised as heterozygous nulls (+/−), with wild-type sequence on one allele and a deletion close to the PAM site on the other.

A further 23 mice had biallelic mutations in *Eef1a2*. Of these, 18 had indels on both alleles, presumably as a result of the unmutated PAM site being targeted by the Cas9 via non-homologous end joining (NHEJ). One animal had an allele with a 21 bp in-frame deletion (denoted **del/−**), but all other alleles were predicted to give rise to premature stop codons and nonsense-mediated decay (mice with two such alleles are categorised as **−**/**−**). Five mice were compound heterozygotes, with a deletion on one allele and clean incorporation of the G70S-causing mutation on the other allele (**G70S/−**). In these cases the G70S mutation had been successfully incorporated by homology directed repair (HDR), but NHEJ had resulted in a deletion on the other allele. Only one mouse was homozygous for the G70S mutation with no evidence for indels (**G70S/G70S**). No mice recapitulated the human clinically relevant G70S/+ genotype, again presumably due to the high levels of NHEJ resulting from the unmutated PAM site (see below).

The remaining six mice all showed clear evidence of mosaicism. Two could not be genotyped with any accuracy as they had complex mutations. In one case, PCR of the region indicated the presence of two mutant alleles, with insertions of approximately 50 bp and approximately 250 bp respectively. In the other mouse (#13) amplification was unsuccessful from the ear notch DNA but subsequent PCR from brain tissue showed a complex pattern of deletions (data not shown). A further four mice were complex mosaics; in two of these there was evidence of G70S incorporation on at least one allele but subsequent analysis showed that the missense mutations were in *cis* with deletions and therefore not expressed.

As shown in the summary of genotyping in Fig. 1C, the G70S mutation was incorporated cleanly in seven alleles and in *cis* with indels on a further 9 alleles, showing that HDR had occurred in 18/71 of alleles that could be sequenced (allowing for mosaicism). The likely reason for the lack of G70S/+ mice was the high frequency of specific deletions in *Eef1a2*. Most indels found were 1–40 bp deletions located close to the 5′ PAM site on the intron/exon boundary (that had not been mutated in the repair template because of the possibility of introducing splicing artefacts). Cas9n makes a nick in DNA 3 bp 5′ to the PAM site, and indeed 34 of the alleles sequenced contain indels either flanking or within 5 bp of the cut site at the intron-exon boundary. The 3′ PAM site, however, was in the coding region, and we had mutated this site in the repair template. In all the alleles sequenced, only two contain deletions that cover this 3′ PAM site, and each of those seems likely to have originated at the 5′ site and extended to the 3′ PAM (Fig. 1C). These results suggest that it could be important to mutate both PAM sites in repair templates in order to minimise subsequent rounds of mutagenesis.

No off-target mutations were detected in any of the mice after amplifying and sequencing DNA around the top 3 predicted off-target sites for each gRNA (data not shown).

### Genome editing of *Eef1a2* produced mice with neurodegeneration and susceptibility to seizures

**Figure 2:**
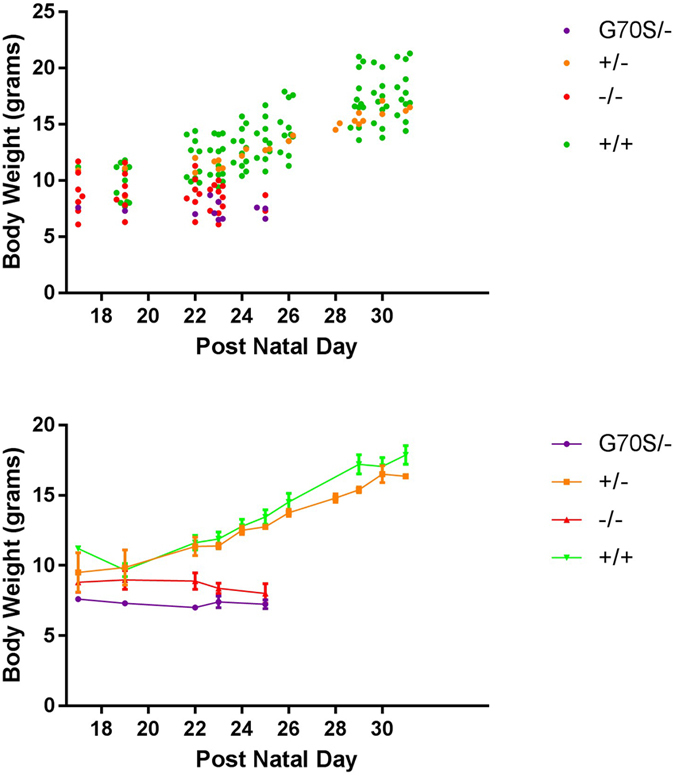
Body weights of F0 mice from weaning. Mouse weights between postnatal day 17 and 30. Upper panel shows data expressed as one dot per mouse per measurement; mouse genotype is indicated by colour of dot. The lower panel shows mean (dot) and standard error at different timepoints for each genotype. Since there was only one WT mouse in the F0 litters, additional body weights for genetically matched control mice were included. Note that the only G70S/G70S mouse is not included in this analysis as it had been culled before weighing began. One-way ANOVA performed on body weights from P23 and, separately, P25, showed that the body weights of mice with biallelic mutations were significantly different from those of +/− and wildtype mice (P < 0.0001 for both P23 and P25). Tukey’s multiple comparison test subsequently showed that the G70S/− and −/− weights were significantly different from wildtype and +/−, on both P23 and P25. Body weights for G70S/− and −/−, and for +/− and +/+ were not significantly different.

All mice were carefully observed from day 14 onwards, weighed regularly, and any abnormal behaviour recorded. A summary of the results is shown in Table 1 and in more detail in Supplementary Table 1; all results were obtained from analysis of founder (F0) mice as no G70S/+ mice were obtained from which to establish breeding lines. Plots of individual mouse weights and growth curves are shown in Fig. 2. Only the four heterozygous null and one wild-type mouse survived. One mouse had no evidence of any mutation in *Eef1a2* but was severely runted at weaning and had to be culled.

#### Mice with biallelic deletions (−/−)

Six mice with clear-cut biallelic deletions in *Eef1a2* had to be euthanised at 23–25 days as they displayed a phenotype typical of an eEF1A2-null “wasted” mouse. This included weight loss after weaning (see Fig. 2), hunched posture, tremors and gait abnormalities (this phenotype is categorised in Table 1 as “wasted”).

Eight mice had seizures in response to transient environmental noise. In the first instance, in response to the sound of liquid nitrogen being poured in a neighbouring room, six 18 day old mice (including one mosaic) had fatal tonic clonic seizures preceded by the wild running typical of an audiogenic seizure. This event occurred before any of the typical phenotypic features of a null mouse would have been observed and is categorised in Table 1 as “seizure”.

In the second instance, two 23 day old mice, both exhibiting a wasted phenotype (and with clear signs of motor neuron degeneration when their spinal cords were examined), had seizures apparently in response to a door banging. One died during the seizure; the other recovered but subsequently had to be culled because of the wasted phenotype (categorised in Table 1 as “wasted + seizure”).

The remaining four mice with biallelic null mutations died suddenly. The mice appeared healthy and were being transferred between animal facilities (which were physically adjoined) but were dead by the time they were received less than an hour later. Given the very low threshold of noise required to induce seizures in the other mice, it is conceivable that the sudden deaths were also the result of audiogenic seizures. As there were so few control mice from this experiment, and the noises were incidental, we can not evaluate the incidence of audiogenic seizures statistically, but no other mice in the facility have ever been observed to have seizures in response to similar environmental sounds.

**Figure 3:**
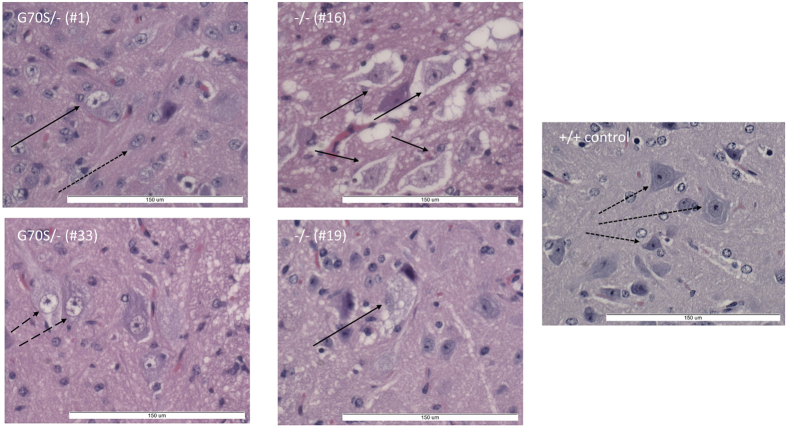
Neurodegeneration in the spinal cords of mice with mutations in eEF1A2. H&E stained sections of of spinal cord taken from G70S/− (left) and null (right) mutant mice, with age matched control section on far right. Normal neurons are indicated by arrows with dotted lines. Dashed line arrows indicate neurons showing nuclear vacuolation and solid arrows indicate degenerating neurons with marked cytoplasmic vacuolation.

Those −/− mice from which spinal cords were able to be obtained showed variable but clear-cut evidence of motor neuron degeneration (Fig. 3). The sections showed a range of degenerative pathology ranging from essentially normal through to striking neuronal nuclear enlargement and cytoplasmic degeneration. An intermediate stage was also seen, with significant cytoplasmic vacuolation but no obvious nuclear changes.

#### Compound heterozygous mice (G70S/−)

Five mice were found to have incorporated the G70S missense mutation cleanly on one allele and to have a deletion on the other allele, with no sign of mosaicism. These mice all appeared to have a phenotype typical of mice that express no eEF1A2. They were indistingishable from their homozygous null littermates and had to be culled at 23–25 days (see Fig. 2).

We were able to analyse spinal cord sections from three of the G70S/− compound heterozygous mice (#1, 10 and 17). All showed clear signs of neurodegeneration (Fig. 3) and the degree of weight loss and overall observable condition were indistinguishable from those of the null mice (Fig. 2).

#### Homozygous G70S/G70S mouse

One mouse exhibited what appeared to be a more severe than usual wasted phenotype. Although mice which are null for eEF1A2 do not show any outward signs of the wasted phenotype until at least 21 days, and normally survive until at least 23–28 days, this mouse had to be euthanised at 18 days. It was smaller than its littermates (most of which were later shown to be complete nulls), and displayed tremors and shakiness. This was the only mouse which was subsequently found to be homozygous for the G70S mutation. This suggests that homozygosity for the missense mutation might result in a more severe effect than that seen in homozygous null animals, but as only one mouse had this genotype this result has to be interpreted with extreme caution.

#### Mosaic mice

Of the remaining mice, five were clear mosaics. The mosaicism is presumed to result from Cas9-induced mutagenesis continuing after the first cell division in the embryo.

One of the mosaic mice died of what appeared to be an audiogenic seizure at 18 days (as described above) and another was found dead at 23 days. The other three mosaic mice also displayed characteristic wasted features of tremor, weight loss and unsteady gait, but two of these survived longer than any typical wasted mouse, until 32 and 35 days respectively (Table 1). A further mouse, #13, which had a complex pattern of deletions on sequencing, survived to 29 days. One of the mosaic mice that had to be euthanised at 23 days had previously had a seizure lasting about two minutes, with repeated rearing and shaking.

#### Expression analysis of eEF1A2 in mutant mice shows the G70S mutant protein is unable to compensate for the loss of wild-type eEF1A2

Tissue samples were collected from all mice with bilallelic or mosaic mutations, and muscle and brain protein extracts analysed by Western blotting with anti-eEF1A antibodies.

**Figure 4:**
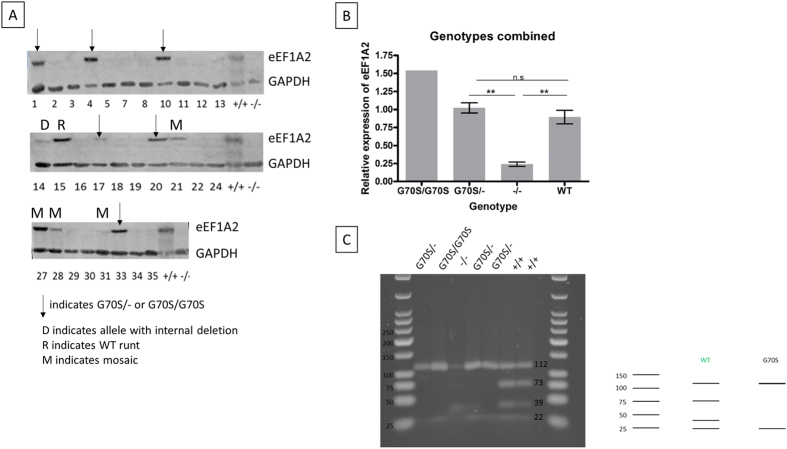
Expression analysis of mice with mutations in eEF1A2. (**A**) Representative western blot showing eEF1A2 expression in protein extracts from brains of mutant founder mice from the CRISPR gene targeting experiment. The negative control was a brain extract from a wasted mouse (denoted −/−) and the loading control was GAPDH. The blot images are cropped for clarity and a larger area is shown in the Supplementary Data File. (**B**) Quantification of eEF1A2 expression in brain samples from mice with key genotypes. Controls were aged matched wild-type mice that were offspring of the F0+/− heterozygous mice. Expression levels are derived from three repeats of the Western analysis, normalised to GAPDH and expressed relative to the average obtained from three independent wild-type controls. **Indicates p < 0.01, n.s means not significant. (**C**) MnlI digests of RT-PCR products from brain RNA of mice of different genotypes. Predicted band sizes are shown to the right of the gel. The absence of bands at 73 bp and 39 bp in the G70S/− and G70S/G70S mice suggests that no wild-type allele is being expressed.

Initially, we used an antibody that was raised against full length eEF1A protein. This antibody does not distinguish between eEF1A1 and eEF1A2, but was used to establish whether any smaller bands were detectable in any samples, that would represent truncated products that could be missed by the isoform-specific anti-peptide antibodies. In fact, no bands were seen in any sample other than the expected band at ~50 kDa (data not shown). An antibody that is specific to the eEF1A2 isoform was then used to examine expression in the same brain extracts from all animals with mutations. Brain samples from all mice carrying a G70S mutation were then run in triplicate together with additional control samples for quantification. Representative blots and a summary of the results are shown in Fig. 4.

Expression of eEF1A2 in brain of mice identified as having biallelic deletion mutations was at background level in all cases, comparable to that seen in tissue extracts from wasted mice (representing complete nulls). Only a trace of expression was seen in brain from mouse #14, that had an in-frame deletion. All mosaic mice apart from #31 (that had to be euthanised at 23 days along with the null mice) had detectable expression of eEF1A2 (Fig. 4A).

Strikingly, mice with the G70S mutation on at least one allele showed levels of expression of eEF1A2 in brain comparable to that seen in wild-type mice (Fig. 4B). Additional analysis was carried out in order to quantify expression in the mutant mice relative to the mean value from three wild-type control samples. All samples were analysed in triplicate. As shown in Fig. 4C brain from G70S/− mice expresses eEF1A2 at levels comparable to those seen in samples from age-matched WT mice. Since the G70S/− mice analysed (#1, 10 and 20) all had clean incorporation of the G70S mutation on one allele and a deletion on the other the protein that was being expressed was predicted to have originated from the G70S mutant allele. Further, mouse #4, a G70S/G70S homozygote also expressed eEF1A2 brain at levels equivalent to wild-type controls.

In order to determine whether the protein being produced in the apparently G70S/− mice did indeed derive from the allele bearing the G70S missense mutation, we carried out RT-PCR on brain samples from all mutant mice carrying a G70S mutation on at least one allele (Fig. 4C). The repair template had been designed such that incorporation would result in the loss of an MnlI site if the G70S mutation was created, allowing us to determine whether any detectable transcript was derived from the mutant allele. The MnlI digest of brain RT-PCR products showed that in each of the mice thought to be compound G70S/− heterozygotes (#1, 10, 17, 20 and 33) and the G70S homozygote (#4) the only allele detectable at the RNA level contained the G70S mutation (Fig. 4C). In the case of G70S/− mouse #20 (not shown), the downstream MnlI site had been lost due to a silent mutation, but the pattern confirmed the presence of the G70S site). This demonstrates that the protein detected by Western blotting is actually the G70S containing mutant eEF1A2, and that even high levels of expression of the mutant protein in brain are insufficient to protect mice from developing a phenotype consistent with that seen in homozygous null mice.

## Discussion

We used CRISPR/Cas9 genome editing to create mice with mutations specifically in the gene encoding eEF1A2, with a high level of efficiency. Whilst a quarter of all alleles recovered contained the desired point mutation encoding the G70S missense mutation, a large number of mice also had deletions. Twenty eight of the 35 mice born after two rounds of injections had mutations on both alleles of *Eef1a2*, and 94% had at least one specific mutation in *Eef1a2*.

Although no mice were obtained that recapitulate the clinically important, neurodevelopmental disorder-causing genotype seen in humans, G70S/+, we did recover a range of mice with phenotypes relating to epilepsy and neurodegeneration. Firstly, fourteen mice displayed weight loss, hunching, tremors and gait disturbances typical of wasted (eEF1A2 null) mice. These mice were typically euthanised at 23 days. A further four mice were mosaics and developed a similar phenotype but survived until up to 35 days.

Of the mice that appeared to have a typical eEF1A2-null phenotype, four were in fact G70S/− compound heterozygotes that were actually expressing the mutant protein in brain at levels comparable to that seen in wild-type mice. We know from earlier work that the eEF1A2-null phenotype is not ameliorated by expression of eEF1A2 in muscle, so lack of expression in brain and spinal cord is responsible for the phenotypic abnormalities^7^. Heterozygous null mice have no apparent motor defects and live a normal lifespan, in contrast to the G70S/− mice that had to be culled by 23 days at the latest. The failure of the G70S mutant protein, even at high levels, to protect mice from developing a phenotype typical of eEF1A2-null animals suggests strongly that the missense mutation renders the protein essentially non-functional, at least in terms of whole animal physiology. Further work is needed to examine function at a biochemical level, but since heterozygous null animals are essentially normal and have never been observed to have seizures, we can be confident that the G70S mutant protein is severely compromised.

There is also a suggestion that the mutant protein could have acquired a level of toxicity reflecting a dominant negative or gain of function mechanism. The phenotype of the only G70S/G70S homozygous animal was more severe than in its nine null littermates, and this animal was euthanised at 18 days (by investigators blind to genotype). However, any result based on a single animal must be regarded as extremely preliminary, and further lines of mice will clearly need to be made in order to establish whether this is a consistent finding, suggestive of a dominant negative or gain of function mechanism.

The second phenotype in the mice relating to the human condition was seen in 8 mice, all with biallelic mutations (one mouse, #8, showed the presence of a wild-type allele on ear clip DNA analysis but must nevertheless have been a mosaic, as it did not express eEF1A2 in either brain or muscle). These eight mice suffered what appeared to be audiogenic seizures that were fatal in all but one case. Two of the 8 animals also exhibited a typical wasted phenotype but the remaining 6 died of their seizures at 18 days, having shown no sign of weight loss, tremors or ataxia. This lack of a neurodegenerative phenotype was not unexpected as wasted mice do not show any of these phenotypic features until at least 21 days^14^. It can be assumed, since none of the mice in this group were expressing eEF1A2, that they would have gone on to develop a neurodegenerative phenotype had they recovered from their seizures. The original line of wasted mice has not been reported to show susceptibility to audiogenic seizures, so it is possible that our F0 null mice were more susceptible due to genetic background differences. Alternatively the random nature of the environmental triggers, coupled with the close observation of the CRISPR-generated mice, could have led to an ascertainment bias.

Four mice died suddenly, three of them within an hour of having been seen, apparently healthy. The cause of death in these cases could not be established. It is possible that the deaths represented sudden death in epilepsy (SUDEP). SUDEP is a known, extreme, consequence of epilepsy responsible for a significant proportion of deaths in individuals with childhood-onset epilepsy. SUDEP has also been seen in a number of mouse models of epilepsy, for example mice with a knock-in of the Q390X mutation in the *Gabrg2* gene, which can suffer SUDEP at any stage^15^ and those with a heterozygous knock-in of N1768D in the *Scn8a* gene; these mice also exhibit seizures and SUDEP^16^. An alternative explanation for the sudden deaths in the eEF1A2-null mice is neurodegeneration of brainstem nuclei leading to respiratory arrest. Again, further lines will be need to be generated, as analysis of F0 mice does not allow for the level of experimental planning required to resolve such issues.

These results represent a potential new example of the emerging links between neurodegeneration and epilepsy. Both SUDEP and audiogenic seizures have been linked to neurodegeneration of the brainstem, and it is well established that uncontrolled seizures and status epilepticus can lead to neuronal death. However, the linking of pathways that can lead to both neurodegeneration and epilepsy has only recently been recognised. It is difficult to establish the effects of loss of eEF1A2 on the brain as mutant animals die as a result of severe neurodegeneration in the spinal cord, but since motor neurons lacking eEF1A2 degenerate and die (presumably due to a failure to carry out *de novo* protein synthesis) it is reasonable to assume that neurodegeneration would also occur in the brain if the mice survived longer. Similarly, the strain of *Gabrg2* mutant mice described above show widespread neurodegeneration in addition to their epilepsy and SUDEP phenotype, in this case caused by accumulation and intracellular aggregation of mutant GABAA receptor subunits^15^. Whilst there is no evidence that intracellular aggregates are forming in the eEF1A2-mutant mice, one of the disease causing mutations in humans, E122K, has been shown to be associated with translational infidelity in yeast^17^, a process that would be predicted to lead to accumulation of misfolded proteins. However, without additional lines it remains possible that there is no functional connection between the seizures and neurodegeneration in our F0 mice.

Both translation elongation factors eEF1A1 and eEF1A2 have been identified at the synapse in transcriptomic and proteomic experiments^18^,^19^. It seems highly probable that *de novo* protein synthesis at the synapse, necessary for synaptic plasticity and memory consolidation, is perturbed in humans with missense mutations in eEF1A2. The most common cause of inherited intellectual disability and autism, Fragile X syndrome, also shows disruption of protein synthesis at the synapse. Whilst in the case of Fragile X syndrome protein synthesis is upregulated, it has been suggested that small changes in synaptic protein synthesis, either up or down, can result in neurodevelopmental disorders^20^. Interestingly, one of the key phenotypic features seen in mouse models of Fragile X syndrome is an increased susceptibility to audiogenic seizures^21^, suggesting the possibility of mechanistic links between disorders caused by mutations in eEF1A2 and FMRP, via a convergence on synaptic protein synthesis. There is no evidence that translation elongation factors have any specificity in terms of mRNAs that are being translated, so any mutational effect would likely be at the level of global protein synthesis.

The G70S mutation is at a highly conserved amino acid adjacent to the eEF1B binding site, so is predicted to show reduced binding to the eEF1B GTP exchange factor. It is also predicted to affect protein stability, but in fact our results suggest that the protein is expressed at levels indistinguishable from that of the equivalent unmutated protein. It is hard to predict with any confidence whether G70S would result in gain or loss of function, but the mutational spectrum of *EEF1A2* suggests that heterozygous loss of function mutations would be either tolerated or lethal, as all clinical mutations identified so far have been missense. In fact no loss of function mutations in *EEF1A2* are seen in the 60 K genomes in ExAC^22^, and *EEF1A2* is one of a few thousand genes judged to be subject to excessive constraint^23^.

Some useful parallels can be drawn between the effects of mutations in eEF1A2 and disorders associated with mutations in tRNA synthetases (since the primary role of eEF1A2 is downstream, to bring aminoacylated tRNAs to the ribosome). Homozygous loss of function mutations in humans in *QARS* (encoding glutaminyl tRNA synthetase) result in severe epilepsy associated with developmental defects in the brain^24^ and similar mutations in *AARS* (encoding alanyl tRNA synthetase) result in myelination defects^25^. On the other hand, mice that are homozygous for an editing-defective mutation in *Aars* exhibit ataxia and Purkinje cell loss accompanied by a build-up of misfolded proteins^26^. One of the epilepsy-causing mutations in *EEF1A2* in humans, E122K, has been shown to be associated with translational infidelity in yeast^17^ and also results in ataxia in patients^11^. Neurons could be particularly vulnerable to the effects of misfolded proteins resulting from failures of proofreading during protein synthesis.

This study is one of relatively few to examine mutant phenotypes in the F0 generation resulting from CRISPR/Cas9 genome editing. In our case we had no choice but to analyse founder mice as the phenotype was fatal in all the animals carrying the desired mutation, and no G70S/+ animals were recovered. Although analysis of F0 mice was not our intended experimental design, such a strategy has actually been proposed on the basis that CRISPR is so efficient that the breeding of animals could be avoided^27^. This methodology is reliant on the absence of consistent off-target effects, low levels of mosaicism, and the availability of sufficient numbers of mice to be able to avoid the confounding effects of rare off-target mutations. Large numbers of mice are also needed where different animals have different indels, as even subtly different deletions can have distinct effects on gene expression and splicing.

A number of recent studies have demonstrated the need for caution in interpreting results based on analysis of F0 animals only. Yasue *et al* used wild type Cas9 and a single gRNA in mice to target *Fgf10*, loss of function of which is known to confer an abnormal limb phenotype. They collected 25 F0 embryos and showed that although 96% had evidence of NHEJ repair, only 56% had limb defects. Four embryos had up to 4 distinct alleles. Thus although mutagenic efficiency was high, analysis was complicated by the presence of both mosaicism and reduced penetrance. Further experiments yielded some embryos with in-frame deletions, another potential complication for interpretation of results^28^. Fujii *et al*, on the other hand, targeted three independent genes in a single experiment, obtaining 9 F0 pups with mutations at all three loci. These pups displayed knockout phenotypes and transmitted mutations to the next generation, arguing against the presence of significant mosaicism^29^. However, small numbers of animals were studied, and it is possible that the risk of off-target mutations would increase with the number of genes being targeted.

One of the few studies to look for off-target mutations at sites not predicted by online CRISPR design tools used exome sequencing of F1 knock-in mice generated using homology directed repair^30^. This study was small, examining DNA from only 4 mice, but one insertion and one deletion was found in one of the mice, and an insertion in a further mouse; two of these mutations would be predicted to be deleterious. By breeding on from these lines, the off-target mutations would in most cases segregate away from the desired mutation so that confounding effects on the phenotype could be identified. Clearly, though, if only F0 animals are being studied and off-target mutations are non-random, phenotypic analysis could be misleading.

One study, on the other hand, made positive use of the mosaicism seen in F0 animals. Zhong *et al* targeted *Kcnj3* (mutations in which cause blindness) using wild-type Cas9 and a single gRNA to generate mutations by NHEJ. Null mutations in *Kcnj3* are lethal postnatally, so that a conditional strategy would normally have to be employed. In the Zhong study, 8 surviving F0 mice showed mosaicism in the retinal pigmented epithelium, enabling the authors directly to compare patches of mutant and WT cells in the eye^31^.

In our experiment, one of very few to examine F0 offspring from an experiment designed to use HDR via a repair template, a number of mice were clearly identified as mosaic because of the presence of more than two distinct alleles in ear-notch DNA. However, it remains possible that in other cases there were lower levels of mosaicism, for example in specific areas of the brain, that could further confound the interpretation of our results. Indeed, one animal showed evidence of low level mosaicism when expression was examined by RT-PCR even though there had been no indication of this when the DNA was analysed. Clearly this level of complication requires large numbers of F0 mice to be generated in order to obtain meaningful results. We saw no mutations at predicted off-target sites; we can not rule out the possibility of apparently random off-target events, but our use of paired gRNAs and the nickase form of Cas9 should have minimised this possibility. Fortunately we were able to retrieve a number of mice with identical mutations where HDR had been successful, allowing us to analyse the effects of the missense mutation on a null background. Our prior knowledge of the homozygous null phenotype was extremely useful in interpreting our results; we saw no real heterogeneity of expression levels in the mice with homozygous deletions other than those explained by mosaicism. It is worth noting, though, that we saw cases where the missense mutation had been introduced by HDR but where there was a deletion on the same allele, presumably due to a subsequent NHEJ effect. This illustrates the need for careful genotyping for correct interpretation of results.

With the caveats described above, our results suggest that the G70 missense mutation in eEF1A2 severely compromises protein function and may even be toxic to the cell. We also show that the presence of biallelic mutations in eEF1A2 in mice can result in seizures and sudden death, providing the first evidence of a link between eEF1A2 and epilepsy in a model organism. There remains, though, an urgent need for animal models that faithfully recapitulate the human heterozygous condition. Without such models it is not possible to be certain whether any therapeutic strategy should be aimed at boosting expression of the mutant form of eEF1A2, or at restoring expression of eEF1A2 to wild-type levels whilst preventing expression of the mutant protein. The efficiency of CRISPR/Cas9 gene editing has put the generation of these animal models within reach.

## Materials and Methods

### CRISPR/Cas9n gene editing

Guide RNA pairs were designed from target sequence of mouse *Eef1a2* around exon 3 using online tools at DNA 2.0 and at http://crispr.mit.edu/. They were synthesised, annealed and cloned into vector px458 from which they were *in vitro* transcribed before microinjection. The sequences were amplified using universal reverse primer and T7 tagged forward primers. The guide RNA PCR template was used for *in vitro* RNA synthesis using T7 RNA polymerase, and the RNA template was subsequently purified using RNeasy mini kit (Qiagen) purification columns. Cas9 (nickase) mRNA was procured from Tebu Bioscience. The repair template was designed and ordered as an ssODN Ultramer from IDT.

Microinjection: samples were prepared and injected into C57BL/6 single cell embryos at the following concentrations: gRNAs 25 ng/ul each, Cas9n mRNA at 50 ng/ul and Ultramer ssODN repair template 150 ng/ul. Prior to the experiment described here, a different gRNA pair was tested and the resulting embryos collected; as only three embryos out of 71 had incorporated the G70S mutation (data not shown) the design was modified in order to achieve higher rates of targeted mutagenesis (Fig. 1).

### Transgenic mouse generation

Mice were housed in the Biomedical Research Facility (BRF) at the University of Edinburgh. All mice were maintained in accordance with Home Office regulations and all protocols had been approved by the local ethics committee of the University of Edinburgh. All methods were performed in accordance with the relevant guidelines and regulations. Embryo transfer was carried out with short term recovery anaesthesia, and analgesia where needed post-operatively. Mutant mice were closely observed for overall clinical condition and were euthanized where necessary to avoid suffering.

Transgenic mice were made using standard pronuclear injection methods of CRISPR reagents into oocytes derived from C57BL/6J mice. There were 35 live born mice as a result of this procedure; those that survived weaning were housed in small single sex groups. Both male and female mice were characterised; both males and females were seen in all groupings according to phenotype/outcome. All phenotyping was carried out blind to genotype.

### Genotyping

Ear notches were taken at weaning, DNA extracted and used for PCR genotyping, initially by restriction digests to detect the presence/absence of the MnlI site destroyed if the G70S mutation had been introduced, and then by direct sequencing. This sequence information was used to design further allele specific PCR/sequencing assays for the 14 mice that had identifiable small deletions that could be used for primer design to selectively amplify the other allele(s). This product could then be sequenced, providing a straightforward method to sequence a single allele without the need to subclone PCR products.

PCR products from eight mice were TOPO cloned either because they hadn’t been suitable for allele-specific sequencing, or because the allele-specific sequencing results indicated probable mosaicism. DNA from G70S mouse #4 was also TOPO cloned for confirmation of genotype, as this was the only likely homozygous knock-in of the cohort.

### Off-target analysis

Genomic DNA around the top off-target sites predicted by the DNA2.0 tools were amplified by PCR and the products sequenced.

### RNA analysis

RNA from brain was extracted using the QIAGEN RNeasy mini kit and DNase digested using the QIAGEN RNase-Free DNase Set. cDNA was then synthesized using the Agilent Technologies AffinityScript Multi Temperature cDNA Synthesis Kit. RT-PCR was performed on the cDNA using primers for eEF1A2 (5′ GCCACGATCAGCACTGCG and 5′ CAAGCGGACCATCGAGAAGT; Sigma). 1 μl of each cDNA sample was added to 24 μl of PCR reaction mix and run on a touchdown PCR program for 24 cycles for eEF1A2 and 30 cycles for actin. Products were checked on a 2% agarose gel. Products were then digested with MnlI or AciI (NEB) and 15 μl each sample run on 2 or 2.5% agarose gels.

### Protein analysis

Protein extracts were made from muscle (hind limb) and brain from each analysable mouse. Tissues were homogenised in 0.32 M sucrose using a bead homogeniser and centrifuged at 10,000 g at 4 °C for 10 minutes. The supernatant was removed and mixed 1:1 with Laemmli buffer. Samples were boiled at 95 °C for 10 minutes, 10% (v/v) DTT added then loaded onto a 10% polyacrylamide gel and run at 120 V for 1.5 hours. The gel was then transferred at 100 V for 1 hour onto a PDVF membrane. Membranes were incubated in 7% acetic acid and 10% methanol for 15 minutes, washed 4 times for 5 minutes in dH_2_O and incubated in SYPRO Ruby stain for 15 minutes. Total protein was then imaged on the Odyssey Fc Imaging System (LI-COR) and membranes were blocked for 1 hour in Odyssey blocking buffer (LI-COR). Blots were incubated with primary antibodies for eEF1A2 (Rabbit; Proteintech; 1:500) and GAPDH (Mouse, Millipore, 1:1000) in Odyssey blocking buffer at 4 °C overnight then washed in PBST 3 × 5 minutes and incubated in IRdye 800CW anti-mouse (LI-COR) or IRdye 680RD anti-rabbit (LI-COR) secondary antibodies at 1:5000 for 1 hour at RT. Images were analysed using ImageJ software to assess band intensity and values expressed relative to the loading control. Differences in expression between mice with the G70S/−, − / − and +/+ genotypes were assessed by one way ANOVA with tukey post-hoc testing.

### Pathology

Spinal cords were collected and fixed from just under half the mice with biallelic mutations together with aged matched C57BL/6 controls. The fixed spines were delcalcified and sectioned. H&E stained sections were examined by an experienced neuropathologist.

## Additional Information

**How to cite this article:** Davies, F. C.J. *et al*. Biallelic mutations in the gene encoding eEF1A2 cause seizures and sudden death in F0 mice. *Sci. Rep.* **7**, 46019; doi: 10.1038/srep46019 (2017).

**Publisher's note:** Springer Nature remains neutral with regard to jurisdictional claims in published maps and institutional affiliations.

## Supplementary Material

Supplementary Information

## Figures and Tables

**Table 1:** Summary of outcomes according to genotype.

Genotype*	wasted	seizure	wasted** + **seizure	found dead	survived to adulthood	other
+/+					1	1 (runt)
+/−					4	
−/−	6	5	2	4		
del/−		1				
G70S/−	5					
G70S/G70S	1^§^					
mosaic	3^¶^			1		
unknown	1					

^*^Determined by genotyping and/or deduced from expression analysis.

^§^Survived to 18 days.

^¶^Survived to 23, 32 and 35 days.
