# Supplementary material for: Biallelic mutations in the gene encoding eEF1A2 cause seizures and sudden death in F0 mice
Source: Sci Rep. 2017 Apr 5;7:46019. doi: 10.1038/srep46019 (PMC5380952; doi:10.1038/srep46019)

Biallelic mutations in the gene encoding eEF1A2 cause seizures and sudden death in F0 mice

Faith C.J. Davies[1], Jilly E. Hope[1,4], Fiona McLachlan[1], Francis Nunez[1], Jennifer Doig[1], Hemant Bengani[2], Colin Smith[3] and Catherine M. Abbott[1,4] *

***Supplementary Figure 1*. Allele specific sequencing of 14 G70S mice.** (A) Each allele-specific forward primer is shown in bold, the sequence obtained from each mouse using that primer shown beneath. Alignments of the alleles identified using basic Sanger sequencing are under each primer, to show how the primer sequence is ablated in the identified allele, forcing the amplification of the uncharacterised allele. (B) Nested PCR results. For each mouse the amplified PCR product(s) for the first round of nested PCR are shown, followed by the smaller second round product, which varies in size depending on the allele-specific primer used and the size of any indels. The wildtype sample shows the first round PCR product, followed by three second-round products, one for each allele-specific forward primer, F1, F2, F3.

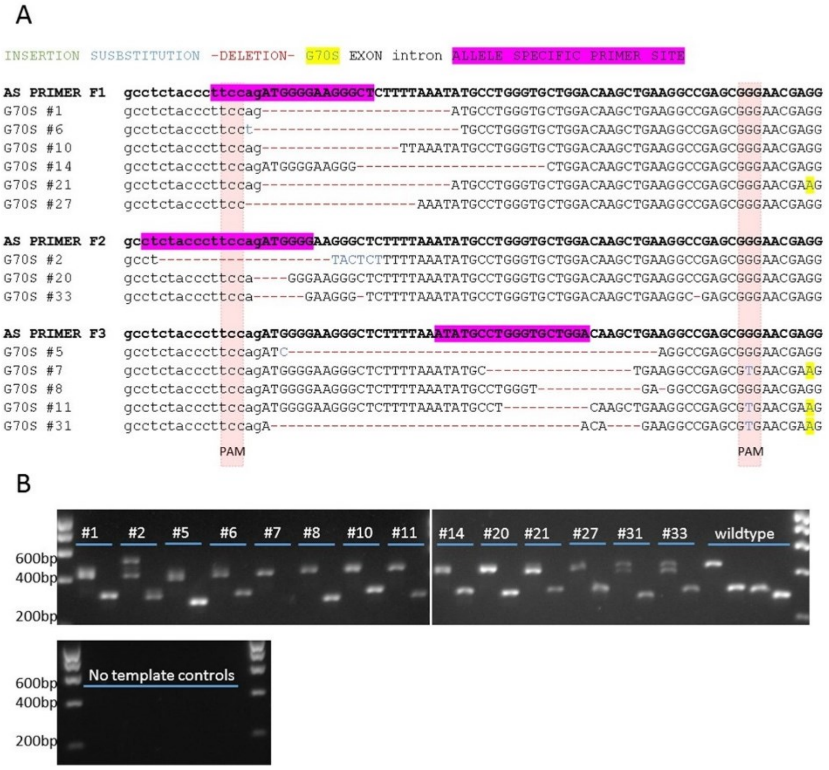

Supplementary Table 1

Mouse number	Sex	Genotype	Phenotype/age at death	Expression
1	M	G70S/-	wasted, 25d	G70S
2	M	-/-	wasted, 22d	null
3	M	-/-	audiogenic seiure, 18d	null
4	M	G70S/G70S	wasted, 18d	G70S
5	M	-/-	audiogenic seiure, 18d	null
6	M	+/-	wild type	nt
7	M	-/-	audiogenic seiure, 18d	null
8	M	+/- ?*	audiogenic seiure, 18d	null
9	M	+/-	wild type	nt
10	F	G70S/-	wasted, 25d	G70S
11	F	-/-	audiogenic seiure, 18d	null
12	F	-/-	wasted, 25d	null
13	F	unknown, complex	wasted, 29d	null
14	F	del/-	audiogenic seiure, 18d	low level
15	M	+/+	runt, culled at weaning	nt
16	M	-/-	found dead, 23d	null
17	M	G70S/-	wasted, 25d	G70S
18	M	-/-	wasted, 25d	null
19	F	-/-	found dead, 23d	null
20	F	G70S/-	wasted, 23d	G70S
21	F	mosaic	found dead, 23d	low level
22	M	-/-	wasted, 23d	null
23	M	+/+	wild type	nt
24	F	-/-	wasted, 23d	null
25	F	+/-	wild type	nt
26	M	-/-	wasted + audiogenic seizure, 23d	null
27	M	mosaic	wasted, 35d	high
28	M	mosaic	wasted, 32d	moderate
29	M	-/-	wasted + audiogenic seizure, 23d	null
30	F	-/-	found dead, 23d	null
31	F	mosaic	wasted, 23d	null
32	F	+/-	wild type	nt
33	F	G70S/-	wasted, 23d	G70S
34	F	-/-	found dead, 23d	null
35	F	-/-	wasted, 23d	null

* Mouse had signs of a WT allele but no detectable expression of eEF1A2 in muscle or brain so effectively null.

nt = not tested

Uncropped Western blots from Figure 4.

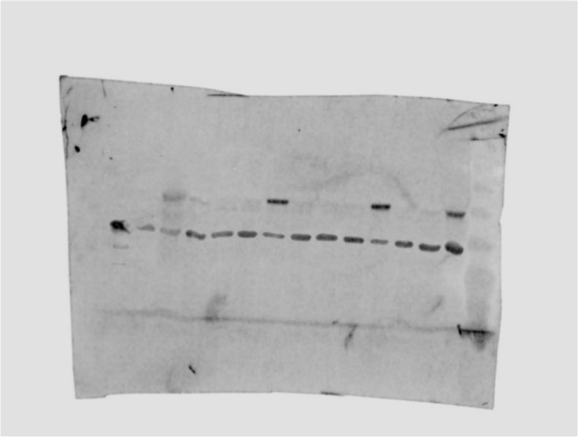

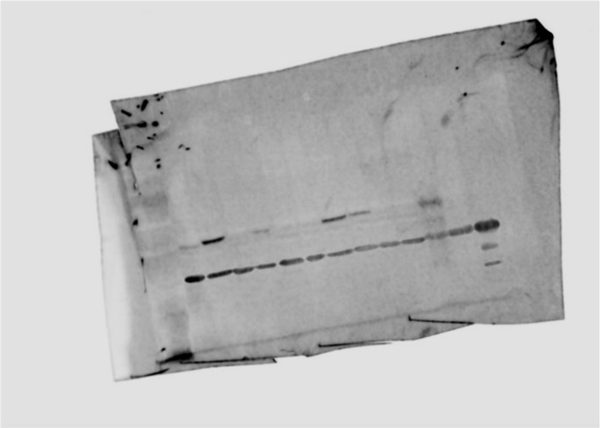

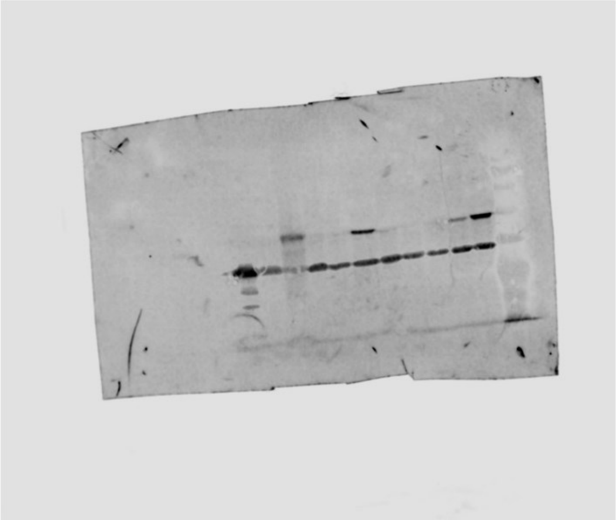

Supplement: Supplementary Information [file srep46019-s1.pdf]
